# The relationships between body composition and cardiovascular risk factors in young Australian men

**DOI:** 10.1186/1475-2891-12-108

**Published:** 2013-08-01

**Authors:** Selma C Liberato, Louise Maple-Brown, Josefina Bressan, Andrew P Hills

**Affiliations:** 1 Menzies School of Health Research, Charles Darwin University, Darwin, Australia; 2 Departamento de Nutrição e Saúde, Universidade Federal de Viçosa, Viçosa, MG, Brazil; 3 Mater Mother’s Hospital/Mater Medical Research Institute, Griffith Health Institute/Griffith University, Brisbane, QLD, Australia

**Keywords:** Blood lipids, Dietary fatty acids, Body composition, Young men, Dietary intake, Cardiovascular risk factors

## Abstract

**Introduction:**

Cardiovascular (CV) disease is a leading cause of global mortality. Despite clear evidence of the coexistence of several risk factors in young people as children and an understanding of the importance of the health behaviors in controlling CV disease, there are limited data on the relationships between risk factors and CV disease in young people. Therefore further study is required.

**Objective:**

This study aimed to investigate associations among body composition, health behaviors and CV risk factors in young Australian men.

**Methods:**

Thirty five healthy men aged 18–25 years had their blood pressure (BP), blood lipids, body composition, resting metabolic rate (RMR), physical activity, dietary intake and cardiorespiratory fitness assessed.

**Results:**

Participants were categorised according to the percentage of body fat into two groups: lean and overweight men. There were no between-group differences in the biochemical indicators except that overweight men had lower HDL-C compared to lean men. Both groups had similar mean energy, protein, fat, carbohydrate and alcohol intake, RMR, physical activity level (PAL) and energy expenditure (EE). Most of the participants (65.7%) had LDL≥2.5 mmol/L. Other common individual risk factors were body fat≥20% (42.9%), waist circumference≥88 cm (28.6%), PAL<1.8 (22.9%) and systolic BP≥130 mmHg (20%). The mean number of CV risk factors was lower among men having a high intake of monounsaturated fatty acids (MUFA, >12% of the energy intake) regardless of whether they were overweight or lean and did not seem to differ according to the source of MUFA consumed.

**Conclusions:**

It is a serious concern to observe such a high percentage of CV risk factors in a group of apparently healthy young men. The likelihood of multiple CV risk factors is greater among those with high body fatness and low MUFA intake. Intake of MUFA favorably affects CV risk factors regardless of the source.

## Introduction

Cardiovascular (CV) disease is a leading cause of global mortality, accounting for almost 17 million deaths annually [1-3]. Twenty five percent of the deaths in 2008 due to non-communicable disease from which CV disease accounted for almost 50%, occurred before the age of 60 [4]. If CV accounts for 37.7% of all deaths [5], almost 10% are likely to be premature. The rate of CV disease is accelerating worldwide and one of the causes is the dramatic increase in the prevalence of obesity with its related complications of hypertension, hyperlipidemia, diabetes and atherosclerotic vascular disease [1]. Any level of overweight appears to increase CV disease risk. The greater the obesity level [6], the body fatness [7] or the abdominal obesity [8], the greater the risk of developing CV disease. Cardiovascular health has commonly been measured by considering the coexistence or clustering of several risk factors in an individual. Traditional CV risk factors include high blood pressure (BP), high serum low density lipoprotein cholesterol (LDL-C), elevated glucose, advancing age, tobacco smoking, male gender, family history of premature CV disease, and other components of the metabolic syndrome such as low levels of high density lipoprotein cholesterol (HDL-C). Other CV risk factors include physical inactivity, low socioeconomic status, elevated psychosocial stress, excessive alcohol and inappropriate diet [3,9-12].

A well balanced diet should contain adequate amounts of protein, fat, carbohydrate, vitamins, minerals, and water. For the maintenance of good nutrition in healthy, normally active persons, there are recommended daily levels of essential nutrients. The largest proportion of the daily energy intake (EI) should be in the form of carbohydrates [13] with approximately 12-20% of the daily EI from protein [13] and 20-35% from fat [13-15]. On the other hand, high intakes of fat (33-40% of EI), particularly monounsaturated fatty acids (MUFA) from olive oil, as in the Mediterranean diet, have been associated with favorable blood lipid profile [14] and reduced predicted coronary heart disease risk in diabetic men [16].

Despite clear evidence of the coexistence of several risk factors in young people as children [17] and an understanding of the importance of the health behaviors in controlling CV disease, there are limited data on the relationships between risk factors and CV disease in young people. In particular, detailed assessment of CV risks and health behaviours in those with early onset overweight may enhance our understanding of premature CV disease risk. Therefore, we sought to investigate associations between health behaviors and CV risk factors in young Australian men aged 18–25 years. We hypothesized that individuals with early onset overweight would display more CV risk factors, fewer healthy and more unhealthy behaviors than those of healthy weight.

## Methods

Thirty five healthy men aged 18–25 y, from the local community in the city of Brisbane, Australia volunteered for the study. Participants were recruited by flyers posted in shopping centers and education centers as well advertisement in local newspapers. Inclusion criteria to participate in the study were age between 18 and 25 years and absence of any chronic disease. Queensland University of Technology Human Research Ethics Committee approved the participant recruitment and data collection procedures. Methods have been previously described in detail [18]. In brief, anthropometric measures including body weight, height and composition, waist circumference (WC) and hip circumference were undertaken. Body mass index (BMI) was calculated as weight (kg) divided by height^2^ (m^2^). Body composition was measured by dual-energy X-ray absorptiometry (DEXA) (DPX-Plus; Lunar Corp, Madison, WI). The participant removed shoes, any materials that could attenuate the x-ray beam, such jewellery, watches and clothes with zippers and laid on his back in the centre of the table. Participants remained motionless in the supine position while the scanning arm of the DEXA passed over their body from head to toe in parallel 1-cm strips. DEXA measurements were made using a constant potential x-ray source of 76 kVp and a cerium filter that produces dual-energy peaks of 38 and 62 keV. The soft tissue mass (fat and lean tissue) is measured pixel-by-pixel as a beam of photons penetrates the participant’s body. Body fat content was determined from DEXA whereas body fat-free mass was calculated by subtracting body fat content from body weight. The DEXA values were used to classify participants into two groups: a) lean (body fat <20%) and b) overweight (body fat ≥20%). BMI >25 and >30 kg/m^2^ correspond to body fat percentage values of approximately 20% and 25% in men, respectively [19-21]. The DEXA values divided by squared height in meters (%body fat mass /m^2^) was also used to classify the participants into groups: lean (body fat/height^2^<6 kg/m^2^) and overweight (%body fat/height^2^≥6 kg/m^2^) [22,23].

Resting metabolic rate (RMR) was assessed by continuous open-circuit indirect calorimetry. A Deltatrac II metabolic cart (Datex-Ohmeda Corp., Helsinki, Finland http://www.hospitalnetwork.com/doc/Deltatrac-II-Metabolic-Monitor-0001) was used to assess RMR of half of the participants. Due to technical problems, the Moxus O_2_ system (AEI Technologies, Pennsylvania, USA) was used to assess the RMR of the remaining participants. RMR measures are less than 100 kcal/d lower using the Deltatrac compared to Moxus according to a study conducted in the same laboratory using the same equipments [24]. A similar proportion of lean and overweight participants were assessed using each of the methods and therefore likelihood of measurement bias was reduced.

Sitting BP was assessed taking one measurement by the same investigator after 10-min rest with a sphygmomanometer. Hypertension was defined as a mean systolic BP>130 mmHg, and ⁄ or diastolic BP>85 mmHg [25]. Following an overnight fast of at least 8 h, a blood sample was collected for total cholesterol (TC), HDL-C, LDL-C and triglycerides (TG) determination using reagents from Roche Diagnostics (Indianapolis, IN). The measurement of TC and LDL-C were based on the determination of Δ^4^-cholestenone after enzymatic cleavage of the cholesterol ester by cholesterol esterase, conversion of cholesterol by cholesterol oxidase, and subsequent measurement by Trinder reaction of the hydrogen peroxide formed [26]. A combination of a sugar compound with detergent was used to selectively determine LDL-C in serum [26]. The HDL-C was determined directly in serum using polyethylene glycol-modified enzymes and dextran sulfate [26]. Low density/high density lipoprotein cholesterol ratio is a risk indicator with greater predictive value than isolated parameters used independently, particularly LDL-C [27]. Total cholesterol<4.0 mmol/L [28], LDL-C<2.5 mmol/L [28], HDL-C>1.0 mmol/L [28], TG<1.7 mmol/L [28] and LDL-C/HDL-C ratio< 3 [27] were target values.

Both food intake and physical activity were assessed over four days. Food intake was assessed by recording household estimates into a food record and entered into the Foodworks (v.3.02) nutrient analysis software (Xris software Pty Ltd. Brisbane, Australia, http://www.xyris.com.au). Macronutrients were expressed as source of energy (percentage of daily EI). Source of MUFA intake was identified and quantified. Physical activity was assessed by physical activity records using nine categories of physical activity intensity (1–9) [29] for each 15-min period throughout the day. The four-day physical activity record scores 1, 2, 3, 4, 5, 6, 7, 8 and 9 correspond to 1, 1.5, 2.3, 2.8, 3.3, 4.8, 5.6, 6 and 7.8 metabolic equivalents (MET) [29], respectively. Using measured RMR, the total daily energy expenditure (EE) was calculated for each participant after accounting for each of the 96 15-min periods of a day and multiplying the score by its specific MET value. Physical activity level (PAL) was calculated by dividing total EE by RMR. Physical activity energy expenditure (PAEE) was calculated by subtracting RMR from EE.

Cardiorespiratory fitness was measured by a continuous speed, incremental grade running test on a treadmill. Participants were fitted with a Polar Coded Transmitter™ and receiver (Polar Electro, Kempele, Finland), a Hans-Rudolf headset (with two-way breathing valve and pneumotach) and a nose clip. After a 4-min warm-up at 3.5 mph, 0% grade, speed was increased to a previously determined comfortable speed, which was the same until the end of the test. Thereafter, the treadmill slope was increased by 2% every min, till the participants reached exhaustion. The rating of perceived exertion using the Borg scale [30] was obtained during each stage and participants were encouraged to achieve a rating of 18 or higher as an indicator of maximal effort. Maximal oxygen uptake (VO_2max_) was assessed using a MOXUS Modular O_2_ System (AEI Technologies, Pennsylvania, USA). VO_2max_ was achieved when the difference between the last 2 completed stages determined by the average of the last 30-sec period before the load increased was <1.6 ml/kg.min or when both heart rate ±10 bpm of 220 – age and respiratory exchange ratio >1.15 were achieved. VO_2max_ was defined as the highest observed value averaged across 15 seconds in a completed stage. When the participant did not reach VO_2max_, VO_2_ peak oxygen uptake, the highest observed value of VO_2_ was considered in analysis. VO_2_ was expressed per kg body weight.

The number of cardiovascular risk factors was calculated by counting how many of the following five factors were present: hypertension (systolic BP≥130 mm Hg and/or diastolic BP≥85 mm Hg [25], high (≥3.0) LDL-C/HDL-C ratio [27], high (≥1.7 mmol/L) TG [31], high (≥88 cm) WC [25], low (<1.8) PAL [32]. National Health and Medical Research Council [13] does not set adequate intake (AI), recommended dietary intake (RDA) or upper limit (UL) values for MUFA. Therefore the median percentage of EI as MUFA (12%) was chosen as a cut-off to divide participants in low and high MUFA intake.

Data are presented as means and standard deviations. The Student’s *t* test was used to compare data. Statistical analysis was performed using Statistic for Windows 5.5 software. A P value less than 0.05 was considered as statistically significant.

## Results

Using percentage of body fat to classify study participants into lean and overweight groups, three participants with a BMI>25 kg/m^2^ were not overweight based on body composition assessment as their body fat percentage assessed by DEXA ranged from 12.5% to 19.2%. Similarly, five participants with a BMI<25 kg/m^2^ had a body fat>20% (data not shown). Participant characteristics are outlined in Table 1. As expected, overweight participants (body fat≥20%) had higher body weight, BMI, circumference measurements and body fat mass than lean participants (body fat<20%). Lean and overweight participants had similar fat-free mass (FFM) levels, but lean participants had proportionally more FFM than overweight participants did. There were no between-group differences in the biochemical indicators except that overweight participants had lower HDL-C compared to lean participants. The mean TC and LDL-C levels were above the target levels of 4.0 mmol/L and 2.5 mmol/L, respectively, even among the lean participants (Table 1).

**Table 1:** Body composition, blood pressure (BP) and blood lipid measures of young men

**Characteristic**	**Lean**^1^**(n=20) **^2^	**Overweight**^1^**(n=15) **^2^	**P values**^3^	**Lean**^4^**(n=18) **^2^	**Overweight**^4^**(n=17) **^2^	**P values**^3^
Age (years)	21.2 (1.7) ^5^	22.5 (2.6)	0.102	20.8 (1.2)	22.7 (2.6)	<0.001
Body weight (kg)	71.1 (10.4)	85.9 (12.2)	<0.001	70.3 (10.3)	85.0 (12.1)	<0.001
Height (m)	1.75 (0.07)	1.78 (0.07)	0.252	1.75 (0.07)	1.77 (0.07)	0.579
Body mass index (kg/m^2^)	23.2 (2.8)	27.2 (3.0)	<0.001	22.8 (2.5)	27.2 (2.9)	<0.001
Waist circumference (cm)	78.3 (2.8)	88.5 (8.1)	<0.001	77.5 (4.7)	88.1 (7.8)	<0.001
Hip circumference (cm)	98.8 (5.9)	107.3 (5.7)	<0.001	97.8 (5.3)	107.3 (5.4)	<0.001
Fat mass (kg)	9.6 (3.8)	22.0 (6.0)	<0.001	9.1 (3.5)	21.1 (6.1)	<0.001
Body fat (%)	13.4 (4.0)	25.7 (5.8)	<0.001	12.8 (3.7)	24.9 (5.8)	<0.001
Fat-free mass (kg)	58.1 (7.7)	60.3 (9.2)	0.448	57.9 (7.8)	60.2 (9.0)	0.419
Fat-free mass (%)	86.6 (4.0)	74.3 (5.8)	<0.001	87.2 (3.7)	75.1 (5.8)	<0.001
Systolic BP (mmHg)	120.3 (9.0)	124.3 (11.2)	0.248	120.7 (9.4)	123.3 (10.8)	0.458
Diastolic BP (mmHg)	57.3 (7.2)	60.1 (7.0)	0.247	56.8 (7.4)	60.2 (6.7)	0.165
TC (mmol/L)	4.33 (0.92)	4.63 (1.06)	0.375	4.26 (0.93)	4.66 (1.01)	0.222
HDL-C (mmol/L)	1.46 (0.29)	1.30 (0.16)	0.043	1.47 (0.31)	1.31 (0.16)	0.082
LDL-C (mmol/L)	2.57 (0.83)	2.78 (0.76)	0.448	2.49 (0.82)	2.84 (0.76)	0.07
LDL-C/HDL-C	1.83 (0.70)	2.20 (0.69)	0.135	1.78 (0.70)	2.21 (0.67)	0.07
TC/HDL-C	3.05 (0.80)	3.62 (0.92)	0.058	3.00 (0.81)	3.60 (0.88)	0.04
Triglycerides (mmol/L)	0.86 (0.30)	1.41 (1.49)	0.172	0.86 (0.31)	1.34 (1.40)	0.167

*TC* Total Cholesterol, *HDL-C* High-density lipoprotein cholesterol, *LDL-C* Low-density lipoprotein cholesterol.

^1^ Lean (%body fat<20%) and overweight (%body fat≥20%).

^2^ Number of participants.

^3^ Probability of means being different by *T* test.

^4^ Lean (body fat/height^2^<6 kg/m^2^) and overweight (%body fat/height^2^≥6 kg/m^2^).

^5^ Standard Deviation.

Using percentage of body fat/m^2^ to classify study participants into lean and overweight groups, two lean participants having % of body fat<20% were classified as overweight because they had %body fat/m^2^ ≥6 kg/m^2^. Participant characteristics are outlined in Table 1. Compared to the results using %body fat to classify the participants into adiposity levels, all body composition, blood pressure and blood lipid results were similar to the results using %body fat/m^2^ except for HDL-C where the difference was no longer significant and for the TC/HDL-C ratio where the higher levels in overweight compared to lean participants became significant (Table 1).

Using percentage of body fat to classify study participants into lean and overweight groups, both groups had similar mean energy, protein, fat, carbohydrate and alcohol intake (Table 2). Carbohydrate contributed less than 50% of EI. Lean participants consumed more energy relative to BW than did the overweight participants. Both groups had similar mean RMR, PAL and EE. Lean participants expended more energy relative to BMI and more physical activity energy relative to body weight than overweight participants. Lean participants had higher cardio respiratory fitness (VO_2max_) compared to overweight participants (Table 2). Four participants had measurements VO_2max_ missing due to technical problems with the equipment or due to inability of the participant to complete the last session of measurements. Most of the participants (65.7%) had LDL-C≥2.5 mmol/L. Other common individual risk factors were body fat≥20% (42.9%), cardiorespiratory level VO_2max_<51 mL of O_2_/min (41.9%), WC≥88 cm (28.6%), PAL<1.8 (22.9%), and systolic BP≥130 mmHg (20%). The least common individual risk factors were TG≥1.7 mmol/L (8.6%), LDL-C/HDL-C ratio>3 (8.6%) and HDL-C<1 mmol/L (5.7%). None of the participants had diastolic BP ≥85 mmHg.

**Table 2:** **Reported food intake, resting metabolic rate (RMR), daily energy expenditure (EE) (kcal/day), physical activity energy expenditure (PAEE) (kcal/day), physical activity level (PAL) and oxygen consumption maximum (VO**_**2max**_**) in young men**

**Characteristic**	**Lean**^1^**(n=20) **^2^	**Overweight**^1^**(n=15) **^2^	**P values**^3^	**Lean**^4^**(n=18) **^2^	**Overweight**^4^**(n=17) **^2^	**P values**^3^
Energy intake^5^, kJ/d; kcal/d	11379 (2249)^7^; 2720 (537)	11172 (2383); 2670 (570)	0.795	11384 (2195); 2724 (525)	10868 (2386); 2600 (571)	0.51
EI/BW, kJ/kg; kcal/d	162.8 (35.1); 38.95 (8.4)	131.4 (29.3); 31.4 (7.01)	0.008	166.36 (7.8); 39.8 (7.8)	130.8 (30.5); 31.3 (7.3)	<0.001
Protein (% of EI)	16.2 (2.4)	17.6 (5.0)	0.334	16.6 (2.2)	17.0 (4.9)	0.72
Fat (% of EI)	32.4 (3.9)	32.0 (6.8)	0.852	32.2 (3.8)	32.3 (6.6)	0.96
Saturated (%)	13.2 (2.6)	12.5 (3.7)	0.520	13.1 (2.4)	12.6 (3.8)	0.67
PUFA (%)	4.5 (0.8)	4.7 (1.2)	0.477	4.5 (0.8)	4.7 (1.2)	0.41
MUFA (%)	12.0 (1.6)	11.7 (2.8)	0.743	11.9 (1.7)	11.8 (2.7)	0.92
Plant/animal ratio	0.80 (0.76)	0.93 (0.86)	0.636	0.8 9 (0.8)	0.92 (0.81)	0.62
Carbohydrate (%)	46.3 (5.8)	48.4 (9.5)	0.688	47.0 (5.6)	48.6 (9.2)	0.52
Alcohol (%)	4.0 (4.9)	2.0 (4.2)	0.201	4.2 (5.1)	2.0 (4.0)	0.17
RMR, kJ/d; kcal/d	7724 (1178); 1846 (283)	8279 (1103); 1979 (264)	0.166	7687 (4924); 1839 (283)	8235 (1108); 1970 (265)	0.17
EE^5^, kJ/d; kcal/d	15051 (2693); 3598 (644)	15212 (2433); 3636 582)	0.856	15214 (2763); 3640 (661)	14992 (2375); 3587 (568)	0.80
EE / BMI^5^, kJ/d.m^2^/kg; kcal/d.m^2^/kg	650 (98); 155 (23)	563 (91); 135 (22)	0.011	666.3 (88.2); 159.4 (21.1)	555.1 (86.9); 132.8 (20.8)	<0.001
PAEE (EE – RMR)^5^, kJ/d; kcal/d	7327 (1739); 1751 (415.6)	6934 (1695.8); 1657 (405.3)	0.508	7524 (1710); 1800 (409)	6755 (1655); 1616 (396)	0.19
PAEE / BW^5^, kJ/d/kg; kcal/d/kg	104 (23); 25 (6)	82 (22); 20 (5)	0.007	107.4 (20.9); 25.7 (5.0)	80.67 (25.7); 19.3 (5.0)	<0.001
PAL^5^	1.95 (0.16)	1.84 (0.19)	0.078	1.97 (0.13)	1.82 (0.19)	<0.001
VO_2max_ (ml of O_2_/kg/min)^6^	55.62 (5.88)	46.30 (5.97)	<0.001	55.8 (6.2)	47.4 (6.2)	<0.001

*EI/BW* Energy intake divided by body weight, *PUFA* Polyunsaturated fatty acids, *MUFA* Monounsaturated fatty acids, *EE / BMI* Energy expenditure divided by body mass index, *PAEE / BW* Physical activity energy expenditure divided by body weight.

^1^ Lean (body fat <20%) and overweight (body fat ≥20%).

^2^ Number of participants.

^3^ Probability of means being different by *T* test.

^4^ Lean (body fat/height^2^<6 kg/m^2^) and overweight (%body fat/height^2^≥6 kg/m^2^).

^5^ Mean of 4 days (2 weekdays, Saturday and Sunday).

^6^ Estimated in 31 (19 lean having %body fat<20% and 12 overweight having %body fat≥20%) participants.

^7^ Standard Deviation.

Using % of body fat/m^2^ to classify study participants into lean and overweight groups, all reported food intake, resting metabolic rate and physical activity results were similar to the results using %body fat to classify the participants into adiposity groups except for PAL where the high PAL of the lean compared to the overweight participants became significant.

**Figure 1:**
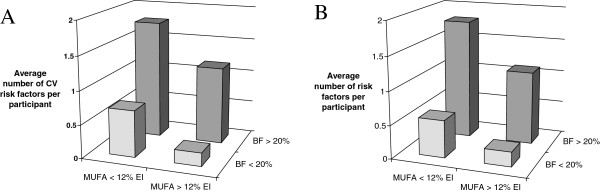
**Average number of cardiovascular (CV) risk factors (including waist circumference >88 cm, physical activity level<1.8, diastolic blood pressure>130 mmHg or systolic blood pressure>85 mmHg, LDL-C/HDL-C ratio>3.0 and triglycerides >1.7 mmol/L) according to body fat (BF) percentage and monounsaturated fatty acids (MUFA) dietary intake percentage contributing to total energy intake in 19 and 16 men consuming low (<12%) and high percentage (>12%) of energy intake as MUFA, respectively. A** - using % of body fat from DEXA to classify study participants into lean (20) or overweight (15) groups. **B** - using % of body fat from DEXA/m^2^ to classify study participants into lean (18) or overweight (17) groups.

Using percentage of body fat to classify study participants into lean and overweight groups, the mean number of CV risk factors was lower among participants having a high intake of MUFA (>12% of EI) regardless of whether they were overweight or lean. The percentage of participants with CV risk factors was higher among those reporting low (<12% of EI) intakes of MUFA compared to those reporting high intakes of MUFA (Figure 1A). Same findings were observed using %body fat/m^2^ to classify the study participants into adiposity levels (Figure 1B).

**Figure 2:**
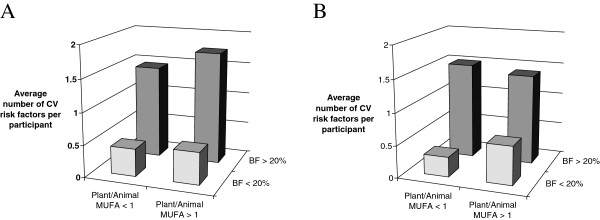
**Average number of cardiovascular (CV) risk factors (including waist circumference>88 cm, physical activity level<1.8, diastolic blood pressure>130 mmHg or systolic blood pressure>85 mmHg, LDL-C/HDL-C ratio>3.0 and triglycerides>1.7 mmol/L) according to body fat (BF) percentage and source of monounsaturated fatty acids (MUFA) dietary intake in 25 and 10 men consuming low plant/animal MUFA ratio (<1) and high plant/animal MUFA ratio (>1), respectively. A** using % of body fat from DEXA to classify study participants into lean (20) or overweight (15) groups. **B** using % of body fat from DEXA/m^2^ to classify study participants into lean (18) or overweight (17) groups.

Using percentage of body fat to classify study participants into lean and overweight groups, the mean number of CV risk factors was comparable between both participants consuming MUFA mainly from plant foods (plant/animal MUFA ratio>1) and participants consuming MUFA mainly from animal foods (plant/animal MUFA ratio<1) regardless of whether they were overweight or lean (Figure 2A). Same findings were observed using %body fat/m^2^ to classify the study participants into adiposity levels (Figure 2B).

## Discussion

A high percentage of CV risk factors, particularly high LDL-C levels and low levels of physical activity and aerobic fitness was observed in this group of young healthy participants. As expected, overweight (body fat≥20%) had higher body weight, BMI, circumference measurements and a greater number of CV risk factors than lean participants (body fat <20%). Most of the parameters assessed were similar between overweight and lean participants except for higher EI/BW, EE/BMI, PAEE/BW ratios and VO_2max_ in the lean compared to overweight participants using either %body fat or %body fat/m^2^ to classify the study participants into adiposity levels. Levels of HDL-C were higher in the lean compared to overweight participants using %body fat to classify participants into adiposity levels but the difference was no longer significant when %body fat/m^2^ was used. Having multiple CV risk factors was more common among those with higher body fatness and lower dietary MUFA intake.

Almost 70% of the participants had high levels of LDL-C and almost half had low levels of cardio respiratory fitness. Aerobic fitness is an important independent predictor of CV disease in middle-aged men. Whereas a lower level of fitness has been associated with a 4.7-fold increased risk of myocardial infarction and stroke [11], moderate fitness seems to protect against the influence of other predictors of mortality in adults [33-35]. Other strong independent risk factors for CV disease include high percentage of body fat [36] and high WC [37,38]. Using 88 cm as a cut-point for WC, 29% of the participants in this study had high WC.

Results in the current study are consistent with published data from other studies with young men for body fat [39,40], RMR [41-44], EE [41,42], PAL [41,42], BP [45-48], VO_2max_[39,49], BMI and circumference measurements [50]. BMI is often used to classify subjects in relation to degree of obesity. However this index has limitations as it does not account for variation in body fat distribution and abdominal fat mass [51]. The misclassification of normal and overweight participants using BMI found in the current study is consistent with a previous study [20]. Participants in the current study had better blood lipid profiles including lower TC, LDL-C and TG and higher HDL-C values compared to other studies examining young men [52-54].

Most of the participants in the current study had one or two CV risk factors. Similar findings have been observed in other studies [55,56]. Populations with multiple behavioral risk factors are at greatest risk for chronic disease and premature death compared with people with single or no risk factors [12,57] whereas participants with CV disease were more likely to have three or more CV risk factors (32%) than those without CV diseases (9.5%) [58]. The higher levels of risk factors at younger ages in men compared to women leads to CV disease presenting 10 years later in women [11].

The prevalence of CV disease has been shown to increase not only with increased number of behavioral risk factors but also with age and obesity level [59]. In addition, obesity was positively associated with the risk of premature death from endogenous causes in populations as young as children supporting the view that childhood obesity is a marker of early metabolic derangement, whereas most of the other risk factors evolve later [60]. Moreover obesity is frequently associated with less favorable biochemical indicators as found in the current study. Lean men in the current study had more favourable biochemical indicators than overweight men. Systolic BP has previously been related to percent body fat [36,61] and obesity, while abdominal obesity and visceral fat have been positively associated with higher TC [7], LDL-C [7,8] and TG [8,62] and negatively associated with HDL-C [62,63]. It has been proposed that the mechanism of the link between increased abdominal fat and risk factors for CV disease is related to higher plasma levels of free fatty acids originating from the enlarged abdominal fat depots [10]. Plasma LDLs cross the vascular endothelium, enter the subendothelial space, become modified and accumulate in the macrophages, which are converted into foam cells that progress to form the atherosclerotic plaque [64]. HDL-C may minimize the accumulation of foam cells in the artery wall and inhibit the oxidative modification of LDL-C [64].

Overweight people have been reported to have higher RMR than lean individuals [65-68]. However, overweight and lean men classified by using either % body fat or %body fat/m^2^ in the current study had similar RMR, possibly due to similar amount of FFM which can contribute up to 80% of the RMR [69].

In the current study, the mean EI, EE and PAEE were similar in both groups and similar to other studies [70,71]. When EI was divided by body weight, the finding of a lower EI per weight observed in overweight men compared to lean men is consistent with other reports [72]. Higher self-reported EI compared to higher self-reported EE for both lean and overweight men suggests underreporting of EI and/or over-reporting of EE which is consistent with other studies [72-74]. This reflects ‘the halo effect’ , where behaviours perceived as ‘good’ will be over-reported (such as doing physical activity), and those perceived as ‘bad’ underreported (such as consuming dietary fat). Also consistent with other studies [66-68], that when EE was divided by BMI and PAEE was divided by BW, these ratios were higher in lean compared to overweight men suggesting that the increased energy cost of moving a larger body mass may have contributed to overweight men expending similar amount of energy compared to lean men.

Lean men in the current study had a higher mean VO_2max_ than the overweight men. Increased VO_2max_ has been associated with increased cardio respiratory fitness. The association between reduced cardio respiratory fitness and increased body fat observed in overweight participants in the current study has been reported in other studies [75-77].

The most striking finding of the current study was that participants consuming a diet in which MUFA contributed a high percentage of EI (>12% of the EI) had an average smaller number of CV disease risk factors compared to those consuming diet in which MUFA contributed to low percentage of EI (<12% of the EI). Dietary MUFA have been found to promote a healthy blood lipid profile, mediate blood pressure, and favorably modulate insulin sensitivity and glycemic control [14]. A reduced predicted coronary heart disease risk by 6.37% was observed in diabetic men consuming a moderately high fat diet (30-50% of EI) in which MUFA accounted for 23% of EI compared to diabetic men consuming a low-fat diet (18-30% of EI) in which MUFA accounted for only 11.4% of the total EI [16]. A systematic review [78] found that dietary MUFA were associated with a 20% reduced risk in coronary heart disease events.

The findings of the current study show that intake of MUFA favorably affects CV risk factors regardless of the source. Favorable effects on blood lipids were found in participants of a randomized parallel controlled-feeding trial undertaken by Bos et al. [79] consuming MUFA predominantly from either plant or animal sources. After a two week run-in diet high in saturated fat, participants were allocated to a high MUFA diet primarily from animal sources, a Mediterranean diet in which MUFA were primarily from plant sources, or the high saturated diet for eight weeks. Participants consuming a high MUFA diet predominantly from animal foods had reduced TC and LDL-C whereas participants consuming a high MUFA diet predominantly from plant foods had increased HDL-C and reduced ratio of TC/HDL-C [79].

Use of body fat estimated by DEXA rather than use of BMI to classify the participants into each group was an advantage of the current study. DEXA is the only widely available technology capable of providing regional measures of fat and lean mass, separating body mass into fat and lean components, thereby permitting the evaluation of fat mass without the confounding influence of other tissue constituents [23]. In addition, it has been shown that fat and lean distribution may predict health outcomes [23]. Studies have shown that lean mass and weight scale with height to approximately the power of two, establishing an analytic framework for height-scaled inces. The use of %body fat/m^2^ has been proposed to classify adiposity levels. However it is not known whether or not the use of the proposed %body fat/m^2^ classification scheme will confer benefits over BMI in terms of predicting obesity-related morbidity or mortality. The %body fat/m^2^ classifications were based on prevalence data, not disease risk, and therefore the clinical utility of the %body fat/m^2^ classification scheme will not be known until data relating disease risk to %body fat/m^2^ becomes available [23]. For these reasons we have used both %body fat and %body fat/m^2^ in order to classify participants of the current study and have showed that there was no difference in the findings using either %body fat or %body fat/m^2^ to classify the study participants into groups.

A small sample size was a limitation of the current study. Another limitation is that RMR of half of the participants was assessed using different equipment due to technical problems. However the likelihood of measurement bias is small because a similar proportion of lean and overweight participants was assessed using each of the equipments. Nevertheless, the results contribute valuable data from a comprehensive clinical assessment of CV risk in young Australian men, an important group which has been under-represented in previous work.

Further studies investigating the effect of MUFA consumption including food sources in CV risk factors in a large sample of broader age range, ethnicity and both genders, are warranted.

## Conclusions

It is a serious concern to observe such a high percentage of CV risk factors in a group of apparently healthy young men. The likelihood of multiple CV risk factors is greater among those with high body fatness and low MUFA intake. Intake of MUFA favorably affects CV risk factors regardless of the source.

## Abbreviations

AI: Adequate intake; BP: Blood pressure; BMI: Body mass index; Cardiovascular: CV; DEXA: Dual-energy X-ray absorptiometry; EE: Energy expenditure; EI: Energy intake; FFM: Fat-free mass; HDL-C: High density lipoprotein cholesterol; LDL-C: Low density lipoprotein cholesterol; MET: Metabolic equivalents; MUFA: Monounsaturated fatty acids; PAEE: Physical activity energy expenditure; PAL: Physical activity level; RDA: Recommended dietary intake; RMR: Resting metabolic rate; TC: Total cholesterol; TG: triglycerides; UL: Upper limit; VO2max: Maximal oxygen uptake.

## Competing interests

The authors declare that they have no competing interests.

## Authors’ contributions

SCL defined the design of the study, undertook data collection, data collation, data analysis and manuscript preparation. LMB helped with the manuscript preparation providing critique and overall scientific input. JB helped with manuscript writing. AH secured support for this study and helped with manuscript writing. All authors read and approved the manuscript.
